# Psychosocial and Psychophysiological Effects of Human-Animal Interactions: The Possible Role of Oxytocin

**DOI:** 10.3389/fpsyg.2012.00234

**Published:** 2012-07-09

**Authors:** Andrea Beetz, Kerstin Uvnäs-Moberg, Henri Julius, Kurt Kotrschal

**Affiliations:** ^1^Department of Special Education, Institut für Sonderpädagogische Entwicklungsförderung und Rehabilitation, University of RostockRostock, Germany; ^2^School of Life Science, University of SkövdeSkövde, Sweden; ^3^Department of Animal Environment and Health, Swedish University of AgricultureSkara, Sweden; ^4^Konrad Lorenz Forschungsstelle GruenauGruenau im Almtal, Austria; ^5^Department of Behavioural Biology, University of ViennaVienna, Austria

**Keywords:** human-animal interaction, animal-assisted interventions, animal-assisted therapy, oxytocin, pet ownership, stress reduction

## Abstract

During the last decade it has become more widely accepted that pet ownership and animal assistance in therapy and education may have a multitude of positive effects on humans. Here, we review the evidence from 69 original studies on human-animal interactions (HAI) which met our inclusion criteria with regard to sample size, peer-review, and standard scientific research design. Among the well-documented effects of HAI in humans of different ages, with and without special medical, or mental health conditions are benefits for: social attention, social behavior, interpersonal interactions, and mood; stress-related parameters such as cortisol, heart rate, and blood pressure; self-reported fear and anxiety; and mental and physical health, especially cardiovascular diseases. Limited evidence exists for positive effects of HAI on: reduction of stress-related parameters such as epinephrine and norepinephrine; improvement of immune system functioning and pain management; increased trustworthiness of and trust toward other persons; reduced aggression; enhanced empathy and improved learning. We propose that the activation of the oxytocin system plays a key role in the majority of these reported psychological and psychophysiological effects of HAI. Oxytocin and HAI effects largely overlap, as documented by research in both, humans and animals, and first studies found that HAI affects the oxytocin system. As a common underlying mechanism, the activation of the oxytocin system does not only provide an explanation, but also allows an integrative view of the different effects of HAI.

## Introduction

During the last decades, animal assistance in therapy, education, and care has greatly increased. Today, the value of animal-assisted interventions [AAI, including animal-assisted therapy (AAT) and activities] is widely acknowledged. In the light of the rapid development of the practice of AAI, research evaluating the effects of AAIs as well as studies investigating the basic effects of human-animal interaction (HAI) and the underlying mechanisms seem to lag behind. Still, there is already quite a body of scientific literature on this topic. However, this is spread out over a number of journals and fields, constraining an integrative view. In the following, we will provide an overview of studies assessing effects of AAI as well as pet ownership which meet certain scientific criteria. In addition, we propose a common underlying mechanism for the majority of the positive effects of HAI: i.e., the activation of the oxytocinergic system and its role in social stress modulation. This system can be linked directly to many of the observed physiological effects of HAI and may also be indirectly associated with the other, mainly psychological, effects. Thereby, we provide a coherent body of theory for integrating the diverse endocrinological, psychophysiological, and psychosocial effects of HAI found in different studies. Furthermore, we give a well-founded overview of scientifically documented effects of HAIs which today are a quite popular topic in the general literature, as are first speculations on underlying mechanisms including the oxytocin system (e.g., Olmert, 2009).

We will start our rationale with a review of the literature on effects of HAI and thereafter we will present a short overview of the oxytocinergic system and its psychophysiological effects. We propose that oxytocin (OT) release may contribute to explain many of the effects of HAI documented by the studies included in our review.

## Criteria for Inclusion of Studies in This Review

In this review we focus on the effects of HAI on psychosocial variables such as empathy and social interactions and on human physical and mental health, including effects on hormones and the autonomic nervous system assessed via variables such as heart rate or blood pressure. Effects could result from either companion animal ownership or animal contacts, in experimental settings or animal-assisted interventions. Studies not directly related to these topics were excluded, for example, on pet ownership and attitudes toward smoking, or other variables which could indirectly influence health or social contacts (e.g., Milberger et al., 2009; Zimolag and Krupa, 2009). Furthermore, we excluded the following contexts: reports on relations between human personality and animal-related issues; the link between animal abuse and interpersonal violence; attitudes toward pets in general or in connection with animal welfare.

The prime criterion for inclusion was the publication of the original research in a peer-reviewed scientific journal. Mainly Medline and PsychLit were used for the article searches. Search terms were: HAI, AAT, animal-assisted activity, “therapeutic riding” and “equine facilitated therapy.” However, since we focus on psychosocial and psychophysiological effects in this review, studies employing horses in physiotherapy with a mere assessment of effects on movement or balance were excluded (e.g., Cherng et al., 2004; Beinotti et al., 2010). From several hundred publications found via the terms “pet,” “cat,” or “dog” we included relevant original research articles (see Table 1) as well as reviews and meta-analyses. The second criterion was that the study design allowed to attribute any effect found with a high probability to the animal interaction rather than to any other possible confounding variable. Hence, studies with a pre-post-measurement design, but without control group were excluded. The third criterion for inclusion was a sample size of at least 10 subjects (per group).

**Table 1:** **Original studies included in the review**.

Authors	Study	Population/age group	*N*	Significant effects of HAI
Allen et al. (2002)	Effect of presence of pets, friends and spouses during a stressor	Adults, married couples	240	People with pets have lower heart rate and blood pressure during baseline than non-pet owners, smaller increases during stressor and faster recovery. Most stress reduction in pet owners when pet was present
Allen et al. (1991)	Effect of presence of pets, friends and alone during a stressor	Adult women	45	Lower blood pressure, heart rate and skin conductance during pet presence
Allen et al. (2001)	Pet acquisition or no pet, stress task	Adults with hypertension	24/24	Lower blood pressure, heart rate, plasma renin activity in the pet group
Banks and Banks (2002)	6 week dog-assisted therapy, control group	Elderly residents in long-term care facilities	45	Reduction of loneliness
Banks and Banks (2005)	6 week dog-assisted therapy in a group setting or individual setting	Elderly residents in long-term care facilities	33	Reduction of loneliness greater in individual setting
Barak et al. (2001)	AAT, non-AAT group	Elderly patients with schizophrenia	10/10	Improved social functioning
Barker and Dawson (1998)	One AAT session vs. one recreational therapy session	Adult psychiatric patients	230	No differences in anxiety between types of session, but significant reduction of anxiety via AAT for patients with different disorders
Barker et al. (2005)	Comparison of petting a dog for 5 or 20 min vs. 20 min resting	Adult health care professionals	20	Reduction of stress, lower salivary and serum cortisol level in the dog conditions
Barker et al. (2003a)	15 min reading vs. 15 min animal interaction before stressor	Adult psychiatric patients	35	Reduction of fear and anxiety
Barker et al. (2003b)	Waiting room with aquarium, waiting room without aquarium before stressor	Adult psychiatric patients	42	Reduction of anxiety
Bass et al. (2009)	12 week therapeutic riding program, waiting list control group	Children with autism	19/15	Greater sensory seeking, sensory sensitivity, social motivation; less inattention, distractibility and sedentary behavior
Beetz et al. (2011)	different groups with social support by dog, adult, or toy dog during a social stressor	Children, age 7–12, with insecure attachment	31	Lower cortisol levels in group supported by dog, strong connection of lower cortisol and physical contact with dog
Berget et al. (2008)	AAT with farm animals, control group; 12 week intervention, 6 month follow-up	Adult psychiatric patients	90	Higher self-efficacy and coping ability in the intervention group, no difference in quality of life
Berget et al. (2011)	AAT with farm animals, control group; 12 week intervention	Adult psychiatric patients	41/28	Lower state anxiety at 6 month follow-up in the intervention group
Bernstein et al. (2000)	Comparison of animal visit and recreational therapy	Elderly residents of two long-term care facilities	33	More initiation of participation in longer conversations
Charnetski et al. (2004)	Experiment: experimental group petting live dog, control groups petting stuffed dog or sitting quietly	Adult college students	55	Only petting live dog increased IgA
Cole et al. (2007)	Different groups, visit with dog, visit without dog, usual care	Adults hospitalized with heart failure	76	Less anxiety, lower blood pressure, lower epinephrine, and norepinephrine levels
Colombo et al. (2006)	Group taking care of canary for 3 months, or group taking care of plant, or group taking care of nothing	Elderly residents living in a home for the elderly	144	Reduction of depression, better quality of life in canary group
Crowley-Robinson et al. (1996)	Comparison of residents of different nursing homes with and without dogs	Elderly nursing home residents	95	Both groups showed less depression
Davis et al. (2009)	10 week therapeutic riding program, control group	Children, age 4–12, with cerebral palsy	35/37	No significant effect on functioning, health, or quality of life
Demello (1999)	Recovery from a cognitive stressor under three conditions: unknown pet absent, present with visual contact only, present with tactual contact allowed	Adult normotensive males and females	50	Reduction of blood pressure and heart rate greater in situation with visual contact only to unfamiliar pet; petting the animal led to lower heart rate
Eddy et al. (1988)	Reaction of strangers when disabled adult is in company of service dog in comparison to being alone	Disabled adults in wheelchairs	10	More smiles and conversations from passersby in presence of dog
Edwards and Beck (2002)	Food intake, weight and need of nutritional supplements before and after introduction of aquarium	Patients with Alzheimer’s disease	62	Increase in food intake, weight, decreased need of nutritional supplements
Fick (1993)	AAT, observation of same group under different conditions, with pet in AAT, without pet	Elderly male nursing home residents	36	More verbal interaction among group members
Fournier et al. (2007)	AAT, control group	Adult prison inmates	48	More social skills and fewer institutional infractions
Friedmann et al. (1983)	Dog present when reading or resting, introduced at beginning or in second half of experiment	Children	38	Lower blood pressure when dog is present from the beginning
Friedmann and Thomas (1998)	Comparison of survival rates of pet owners and non-owners, no intervention	Adult patients with myocardial infarction	424	Higher survival rates of pet owners
Gee et al. (2010a)	Match-to-sample tasks in presence of dog, stuffed dog, or human	Children in pre-school	12	Fewer irrelevant choices or errors in the real dog condition
Gee et al. (2010b)	Memory task in presence of dog, stuffed dog, or human	Children in pre-school	12	Fewer prompts needed in real dog condition, most prompts in the human condition
Gee et al. (2007)	Performance of motor skill tasks in presence or absence of dog	Children, typical and developmentally delayed	14	In dog presence faster completion of the task
Gee et al. (2009)	Dog present or absent during task	Children in pre-school, with or without language impairment	11	Better adherence to instructions in presence of the dog in the imitation task
Grossberg and Alf (1985)	Stroking a dog vs. reading, resting, chatting	Adult students	48	Lower blood pressure when stroking a dog vs. reading, or chatting; link to positive attitudes toward pets
Gueguen and Cicotti (2008)	4 Experiments, experimenter with dog or no dog present, eliciting help or phone number from others	Adult strangers	80	More helping behavior and more trust (giving stranger own phone number) in company of dog
Handlin et al. (2011)	Experiment: stroking the own dog and talking to it for 3 min, control group without dog interaction	Female dog owners (age >30 years)	10/10	Lower heart rate 55 min after interaction in dog and owner, oxytocin levels higher during or shortly after interaction with the dog
Hansen et al. (1999)	Physical examination; one group in the presence of an unfamiliar dog, control group without dog present	children, age 2–6, 14 male, 20 female	15/19	No significant differences in blood pressure, heart rate or fingertip temperature; lower behavioral distress in the dog group
Hart et al. (1987)	Social acknowledgment by strangers before and after acquiring a service dog, comparison to group without dog, self-report	Adults in wheelchairs	19/9	More friendly social acknowledgment since having a service dog, more social interaction than no-dog group
Haughie et al. (1992)	Live pet presence group vs. photography group	Elderly psychiatric inpatients	37	More desirable social interaction
Havener et al. (2001)	Undergoing a dental procedure either in presence of a dog (20) or no dog (20; subgroup of 17 children with self-reported stress)	Children, age 7–11	20/20	No differences between group in peripheral skin temperature; in subgroup of children with self-reported stress (*N* = 17) dog presence decreased arousal while waiting for the dentist
Headey et al. (2008)	Repeated survey comparing pet-owners and non-owners, no intervention	Normal adults	10,969	Fewer self-reported doctor visits, better health
Hergovich et al. (2002)	Children with classroom dog, control class without dog	Children, first grade	46	More empathy, more field independence, more social integration, less aggression
Holcomb et al. (1997)	Presence or absence of an aviary in the center (ABAB design)	Elderly males in day health care program	38	No difference in depression due to mere presence of absence of aviary, but utilization of aviary was associated with lower depression
Jenkins (1986)	Stroking own dog vs. reading aloud	Adults	20	Lower blood pressure while stroking dog
Jessen et al. (1996)	Having a companion bird for 10 days after admission to rehabilitation unit, or no bird	Older adults	20/20	Decrease in depression in the group with a companion bird
Kaminski et al. (2002)	AAT vs. play therapy	Children, hospitalized	70	Both groups had reduced depression, only AAT group had improved in positive affect and mood, lower heart rate
Kotrschal and Ortbauer (2003)	Observation of children in classroom first without dog, then with a dog	Children, first grade, mostly immigrant background	24	Better school attendance, more social integration, less aggression, more attention toward teacher
Kramer et al. (2009)	Visit by person only, by person with dog, by person with robotic dog	Female nursing home residents with dementia	18	More social interaction in presence of real dog and robotic dog than of person only. Robotic dog induced longer looks and conversation
Lang et al. (2010)	Clinical interview in presence of absence of a dog, cross-over design, pre-post measurement	Adult patients with acute schizophrenia	14	Decreased state anxiety after interview in dog presence
Marr et al. (2000)	AAT, control group	Adult psychiatric inpatients, age 20–66	69	More interactive with other patients, more smiles, more sociable and helpful
Martin and Farnum (2002)	Interaction with toy, stuffed dog, or live dog	Children with pervasive developmental disorders, age 3–13	10	More playful, more focused and more aware of social environment
Miller et al. (2009)	Interaction with dog, or reading without contact to dog	Male and female adult dog owners	20	Serum oxytocin increased in women when interacting with their dog, but not in men
Motooka et al. (2006)	Comparison of two conditions: Walking with or without unfamiliar dog for 30 min	Elderly citizens, age 62–82	13	Heart rate variability is higher when walking with the dog than when walking alone
Na and Richang (2003)	Survey comparing pet-owners and non-owners, no intervention	Normal, empty-nester families, adults with grown up children	719	Self-reported better mental and physical health, more still married
Nagasawa et al. (2009)	30 min interaction with dog. Observing gaze from dog to owner, vs. interaction with owner who is not looking directly at dog during interaction	Adult dog owners	55	High attached owners with longer gazes from their dogs had higher OT level in urine after interaction, but not in control condition (not looking at dog)
Nagengast et al. (1997)	10 min standardized physical examination in presence or absence of friendly unfamiliar dog; cross-over design	Children, age 3–6	23	More decrease in systolic blood pressure and heart rate during the examination in the presence of the dog
Nathans-Barel et al. (2005)	AAT, 10 weekly sessions, no AAT group	Adult psychiatric patients with chronic schizophrenia	10/10	Improved hedonic tone, better use of leisure time and higher motivation
Odendaal (2000); (Odendaal and Meintjes, 2003)	Petting own or unfamiliar dog or reading book	Adults	18	Decreased cortisol in human, increase in b-endorphin, oxytocin, prolactin, phenylacetic acid and dopamine in dog and human
Paul and Serpell (1996)	Group with new family dog, group without new family dog	Children, age 8–12	27/29	More visits by friends, more family activities together
Prothmann et al. (2006)	Therapy session with dog, control group without dog	Children, psychiatric patients	61/39	Improvement of vitality, intra-emotional balance, social extroversion and alertness
Prothmann et al. (2009)	Interaction with dog, person, or object	Children with autism	14	Longer and more interaction with dog
Sams et al. (2006)	Occupational therapy with and without dog	Children with autism, age 7–13	22	More language use and social interaction in dog condition
Schneider and Harley (2006)	Watching video of psychotherapists with and without dog present in the video	Adult students	85	More self-disclosure and satisfaction with therapist with dog
Shiloh et al. (2003)	Experiment, exposure to stressor, then stroking different animals or resting	Non-clinical adults	58	Stroking live animals reduced anxiety
Straatman et al. (1997)	Presence or absence (control group) of unfamiliar dog during stressful speech task	Male adults	17/19	No significant difference in anxiety, heart rate or blood pressure between groups
Viau et al. (2010)	Before and after introduction of service dog to family, and after it was removed for short time	Children with autism spectrum disorder	42	Diminished cortisol awakening response after introduction of service dog, this changed back after removal; no effect on average diurnal cortisol level; parents report fewer problematic behaviors when dog was present
Villalta-Gil et al. (2009)	AAT group, control group	Adult inpatients with chronic schizophrenia	12/9	More social contact, fewer symptoms, better quality of life in AAT group
Vormbrock and Grossberg (1988)	Visual vs. verbal vs. tactile interaction with a dog	Adult students	60	Blood pressure lowest while stroking the dog, lower blood pressure when talking to dog than when talking to experimenter, lower heart rate when quietly touching dog
Wells (2004)	Woman in company of different dogs or neutral stimuli in public, reactions of strangers	Adults, strangers	1800	More social acknowledgment from strangers in company of dog
Wesley et al. (2009)	AAT group, control group	Adults with substance abuse	135/96	Better therapeutic alliance with therapist
Wilson (1991)	Comparison of three conditions: reading aloud, reading quietly, interacting with a dog	Young adults	92	No difference in state anxiety between reading quietly and interacting with dog, but both differed from reading aloud

## Effects of Human-Animal Interaction

### Effects on social interaction

Interacting with animals influences social interaction between humans and related factors important in this respect, such as trust, empathy, aggression, and a positive mood.

#### Increased positive social attention from others and stimulation of social behavior

A relatively large body of research investigated the effect of a friendly animal on the perception of the human in its company and on the stimulation of social behavior. This is also called the “social catalyst effect” when it refers to the facilitation of interpersonal interactions.

Hart et al. (1987) and Eddy et al. (1988), for example, showed that the company of a service dog promoted friendly social attention, smiles, and conversation from others for persons in wheelchairs. Wells (2004) studied the behavior of 1800 strangers toward a female experimenter in six different conditions: accompanied by a Labrador retriever pup, by an adult Labrador, by an adult Rottweiler, being in the presence of a teddy bear or a plant, or being alone as control conditions. In the alone condition, the experimenter was ignored more than with the teddy or plant, but got more attention in the company of a dog. The Rottweiler led to more non-responses than the pup or the adult Labrador, which elicited most smiles and verbal responses. Also, in a classroom of first-graders, the presence of a dog was associated with increased attention toward the teacher in comparison to class in the absence of the dog (Kotrschal and Ortbauer, 2003).

Interaction with an animal is *per se* a form of social behavior. The following research assessed the effect of animal presence on this aspect without further investigating effects in interpersonal behavior. Children with autism interacted most frequently and for the longest periods with a real dog in comparison to objects or a person (Prothmann et al., 2009). Also children with pervasive developmental disorders (including autism) were more playful in interaction with a live dog compared to toys, and also more aware of their social environment in the presence of the dog (Martin and Farnum, 2002).

The following studies focused on the facilitation of interpersonal interaction by the presence of an animal, the social catalyst effect. Among children with autism the presence of a dog during occupational therapy was associated with greater use of language and more social interaction (Sams et al., 2006). Similarly, therapeutic riding enhanced social motivation of children with autism (Bass et al., 2009) and in children with various psychiatric diagnoses psychotherapy sessions supported by the presence of a dog promoted social extroversion (Prothmann et al., 2006).

In adult patients with chronic schizophrenia dog-assisted therapy was linked to improvement in social contact, symptoms, and quality of life related to social relationships, but in comparison to a control group without a dog differences were not significant (Villalta-Gil et al., 2009). In psychiatric inpatients, however, AAT lead to a significant increase in interactions with other patients over the course of 4 weeks in comparison to rehabilitation without animals. This included smiles, sociability, helpfulness toward others, activation and responsiveness (Marr et al., 2000). Many animal-assisted interventions focus on elderly residents or patients. The presence of an animal positively influenced, e.g., social interaction in elderly psychiatric inpatients (Haughie et al., 1992) and verbal interaction among male nursing home residents (Fick, 1993). A comparison of observations of AAT and non-AAT recreational sessions in long-term care facilities showed that the animal involvement was linked to more frequent initiation and longer durations of conversations (Bernstein et al., 2000). Kramer et al. (2009) also investigated visits by a person alone, in the company of a dog, and in the company of a robotic dog (AIBO) in female nursing home residents with dementia. The visit of a person with a live dog as well as a robotic dog led to more social interaction than the person alone. From their reviews on the effects of animals-assisted therapy on patients with dementia, Filan and Llewellyn-Jones (2006) and Perkins et al. (2008) concluded that AAT can benefit these patients by increasing social behavior and interaction.

Several studies assessed changes in social interaction not via direct observation, but rather indirectly. Paul and Serpell (1996) found that normal families who obtained a dog, 1 month later engaged in more leisure activities together and their children were more often visited by friends. In a classroom of first-graders, the presence of a dog led to a better social integration among students as documented via indirect psychometric indicators (Hergovich et al., 2002) as well as via direct behavior observation (Kotrschal and Ortbauer, 2003). Also adults profit from animal contact with regard to social relationships, such as patients with substance abuse in an AAT group program, who rated the therapeutic alliance with the therapist as more positive after 26 sessions than the control group without an animal present (Wesley et al., 2009). Fournier et al. (2007) reported that prison inmates significantly improved in social skills via AAT and in normal adult couples, owning pets stabilized the marriage after the children had left home (Na and Richang, 2003).

Based on the presented evidence we conclude that contact with companion animals holds the potential to promote social interaction and functioning in children and adults with or without mental health problems.

#### Increased trust and trustworthiness

Two studies meeting our criteria assessed whether the presence of a friendly animal would increase trust toward other humans. Schneider and Harley (2006) asked college students to rate the trustworthiness of two different psychotherapists, each of them depicted once with a dog present and once without the dog in a video. When the dog was present, participants, particularly those with the least positive attitude toward psychotherapists, reported more general satisfaction with the therapist as well as more willingness to disclose personal information. Gueguen and Cicotti (2008) investigated the influence of the presence or absence of a dog on social interaction, helping, and courtship behavior. In four different experiments, experimenters asked strangers for money in the street, young women for their phone numbers in public or observed whether people would help to pick up coins a male experimenter dropped on the street. The presence of the dog was linked to a higher compliance with the request for the phone number and a higher rate of helping behavior. In particular the compliance with the request for the phone number can be interpreted as an indication for increased trust and maybe also attraction of the strangers toward an unfamiliar man accompanied by a dog, which probably promoted his perception as a trustworthy person. These first findings indicate a sociopositive effect of dogs on trust and prosocial behavior, but clearly, more research is needed.

#### Effects on empathy

Most studies on empathy and animal ownership are designed in a way that they are not conclusive with regards to a direct influence of pet ownership on the development of better empathic skills. For example, Poresky and Hendrix (1990) assessed empathy in young children via reports of their mothers and found that the mere presence of a pet in the household was unrelated to empathy, while the bond with the pet was positively related to empathy and social competence. However, as the authors point out, it cannot be deducted from such a survey-design that the effect is due to the animal. This also applies to the studies by Paul (2000) or Daly and Morton (2003, 2006, 2009). However, Hergovich et al. (2002) documented a positive effect of the presence of dogs in the classroom on the development of empathy in children. When compared to a control class, the class with the dogs showed higher scores in field independence and empathy toward animals. Field independence was interpreted as an indicator of better empathy, since it assesses one’s ability to distinguish between self and non-self, which is a necessary prerequisite for sensitivity toward the moods and needs of others. Clearly, more research with appropriate designs and measures is needed to provide evidence for an effect of animal contact on empathy.

#### Reduction of aggression

Only few results point to the potential of the presence of a friendly animal to reduce aggression in humans. In two studies, effects of the presence of friendly dogs on aggressive behavior in a classroom of first-graders were investigated via behavior observation and reports of the classroom teacher (Hergovich et al., 2002; Kotrschal and Ortbauer, 2003). In the presence of the dog, in comparison to its absence, aggressive behavior was decreased.

#### Reduction of depression and promotion of a positive mood

In their meta-analysis Souter and Miller (2007) conclude that animal-assisted interventions have the potential to significantly reduce depressive symptoms and also our present survey of the literature meeting our criteria points in this direction.

Crowley-Robinson et al. (1996) found a decrease in depression over the course of 2 years in elderly residents of a nursing home with a resident dog, but also in the home without a resident dog. Banks and Banks (2002, 2005) showed in two controlled studies with patients in long-term care facilities that animal visitation programs reduced feelings of loneliness. The effect was stronger in individual dog visits than in group settings, probably since persons had more intense interactions with the dogs in an individual setting. This indicates also that animal visits reduce feelings of loneliness *per se*, instead via facilitating social interactions with the other group members. Elderly residents of an institution experienced a reduction in depression and improvement in quality of life when caring for a canary for a period of 3 months (Colombo et al., 2006). A companion bird also reduced depression in elderly adults after admission to a skilled rehabilitation unit (Jessen et al., 1996). While with one’s own bird individual interaction and stroking are possible, this is different in aviaries which do not allow for direct contact. However, while the mere presence of an aviary did not significantly affect depression in a Veteran’s Medical Center, the intensity of use of the aviary by the elderly men was associated with reduced depression (Holcomb et al., 1997).

Also in children and adults with physical or mental health problems animal contact can improve mood. Nathans-Barel et al. (2005) found that a 10-week AAT-program for patients with chronic schizophrenia improved the mood in comparison with a group without AAT. Children with psychiatric disorders showed better intra-emotional balance after only a single therapy session with a dog (Prothmann et al., 2006). In hospitalized children, both, AAT and traditional play therapy improved mood, as reported by the parents and children themselves, but only AAT was associated with display of positive affect (Kaminski et al., 2002).

### Anti-stress effects

A large body of studies investigated the effect of interacting with animals on stress, operationalizing stress either via endocrinological or cardiovascular parameters.

#### Effects of HAI on cortisol, epinephrine, and norepinephrine

HAI has been investigated for its effects on hormonal indicators of stress such as cortisol, and on neurotransmitters such as epinephrine and norepinephrine. First, studies not employing a specific stressor, then studies including a stressor are reported. These studies provide direct evidence that interaction with a friendly companion animal, in particular a dog, positively affects endocrine responses as indicated by changes in the levels of cortisol, epinephrine and norepinephrine, suggesting an attenuation of stress responses via HAI.

Barker et al. (2005) compared the effects of 20 min of quiet rest to 5 and 20 min interaction with a therapy dog in healthcare professionals. Before (baseline), during and after the interaction or resting, serum cortisol, epinephrine, and norepinephrine as well as salivary cortisol were collected. A significant reduction of serum and salivary cortisol, but no effects on the other parameters, were found in the dog conditions. Odendaal (2000) and Odendaal and Meintjes (2003) assessed changes in plasma cortisol in dog owners when petting their own, or an unfamiliar dog, or quietly reading a book. The interaction with their own dog, and also with the unfamiliar dog, but not the reading condition led to a significant decrease in the cortisol levels of the humans. Viau et al. (2010) investigated cortisol levels of children with autistic-spectrum disorder before and after the introduction of a service dog into their families and after the dog was removed for a short period of time. While no change in the average diurnal cortisol levels due to the intervention or the dog’s removal was observed, the change in cortisol levels after waking up (cortisol awakening response) dropped significantly from 58 to 10% in the morning when the dog was present in the family, and increased back to 48% upon removal of the dog.

A study by Cole et al. (2007) compared a visit with a dog to a visit without a dog and the usual care in the hospital as control conditions among adults hospitalized with heart failure, which can be seen as a naturally occurring stressor. Significantly lower epinephrine and norepinephrine levels were measured during and after the dog visits. The effect of social support by a dog in comparison to support by a friendly human during a social stress test on the cortisol levels of children with insecure attachment representations was investigated by Beetz et al. (2011). The support by a friendly dog during the experiment was associated with significantly lower cortisol levels than support by a friendly human. This effect was strongly correlated with the time the children spent in physical contact with the dog during the experiment.

#### Effects on blood pressure, heart rate, and heart rate variability

A substantial number of well-designed studies investigated the effect of HAI on blood pressure and heart rate, some also included skin temperature or skin conductance, either in the absence of a specific stressor or during a stress-inducing task.

Friedmann et al. (1983) investigated the effect of the presence of a dog on children while they were reading or resting. Blood pressure was lower when the dog was present during the entire time than when the animal was just introduced during the second half of the observation time. Grossberg and Alf (1985) compared the effect of stroking a dog vs. resting, chatting, or reading in undergraduate students. Blood pressures were significantly lower when stroking a dog than when chatting or reading, however, it was lowest during rest. A positive attitude toward companion animals was associated with lower mean arterial pressure and systolic blood pressure. Vormbrock and Grossberg (1988) assessed heart rate and blood pressure while undergraduates interacted with a dog visually, verbally, or tactually. Blood pressure was highest while talking to the experimenter and lowest during stroking the dog. Kaminski et al. (2002) found that in AAT heart rate of hospitalized children decreased and a display of positive affect increased, while this was not the case in play therapy. In adults hospitalized with heart failure, a 12-min visit by a person with a dog led to a greater decrease in systolic pulmonary artery pressure during and after the visit when compared to a visit by a person alone (Cole et al., 2007). Motooka et al. (2006) employed heart rate variability as a parameter associated with autonomic nervous system arousal in healthy elderly adults walking with or without an unfamiliar dog for 30 min. While walking the dog, heart rate variability was significantly higher than when walking alone. Generally, higher heart rate variability indicates a relaxed state and an increase of parasympathetic activity. Jenkins (1986) found that blood pressure was significantly lower when stroking one’s own dog at home than while reading aloud. Similarly, Handlin et al. (2011) showed that stroking ones’ own dog for just 3 min led to decreased heart rates 55 min later in female dog owners, while no such response was observed in a control group not petting a dog.

The following studies assessed the effect of interactions with unfamiliar animals on heart rate and blood pressure before, during or after a stressor. Nagengast et al. (1997) found heart rate and systolic blood pressure of 3–6 year-old children during a standardized physical examination as a mild natural stressor to decrease more in the company of a friendly dog than when undergoing this examination alone at another time. In a similar study, Hansen et al. (1999) compared blood pressure, heart rate, and fingertip temperature between two groups of 2–6 year-old children undergoing a standard physical examination, one group in company of a friendly dog present during the examination, the other group without a dog. Behavior observation documented less behavioral distress when the dog was present. However, there were no significant differences in the physiological parameters between the two groups. Havener et al. (2001) studied the peripheral skin temperature as indicator of autonomic nervous system arousal during a dental procedure in children age 7–10. The intervention group had a dog beside them during the procedure, while children in the control group had no dog or a supportive person present. Only children who had stated before the procedure that they were stressed by having to come to the dentist showed a significant attenuation of the stress response, measured as less decrease in skin temperature; the presence of the dog was observed for the time while the children waited for the dentist to arrive. At the group level, the intervention group did not significantly differ from the control group. Demello (1999) studied adults while they were recovering from a cognitive stressor under three conditions, with a pet present and only visual contact allowed, or with tactile contact allowed; in the third condition no pet was present. As expected, the cognitive stressor led to an increase in heart rate and blood pressure and these parameters decreased most in the condition where the pet was present but no tactile contact was allowed. Stroking the animal did not affect blood pressure, but resulted in a significant reduction of heart rate. Straatman et al. (1997) however, found no effect on heart rate and blood pressure in a group of male students who had a friendly, unfamiliar dog present during a stressful speech task in comparison with a control group without a dog.

Studies investigating the effect of an aquarium in the room differ from others due to the species and restricted possibilities for contact. In a sample of elderly adults, DeSchriver and Riddick (1990) compared the effects of watching an aquarium, a fish videotape, or a control tape on heart rate, skin temperature, and muscle tension. While the group observing the real aquarium showed a trend for lower heart rate and muscle tension as well as for an increase in skin temperature, none of the group comparisons reached statistical significance. Similarly, Barker et al. (2003b) found no significant effect of an aquarium on heart rate or blood pressure in psychiatric patients waiting for an electroconvulsive therapy session.

The effect of the presence of one’s own pet vs. the presence of a friend or being alone on heart rate, blood pressure, and skin conductance during an arithmetic, stress-inducing task performed at home and in the laboratory, was studied by Allen et al. (1991). At home, pulse rate, blood pressure, and skin conductance were lower in the presence of the pet than when alone or with a friend. Allen et al. (2002) investigated the effect of two stressors (an arithmetic task and putting the hand into ice-water for 2 min) in married couples, either in the presence of a friend, their own pet, or the spouse. In the presence of their pets, pet owners showed significantly lower heart rate and blood pressure before the task, less increase in reaction to the stressor and a faster recovery than the non-pet-owning participants who had a friend present. Among pet owners the presence of the pet attenuated the stress response more than the presence of the spouse. Hypertensive patients profit from acquiring and having a pet with regard to stress-related parameters such as heart rate blood pressure and plasma renin activity, an indicator of hypertension. In a study by Allen et al. (2001) all hypertensive participants showed similar reactions toward a mental stressor before acquiring a pet. Then all participants started to take medication for hypertension and half of the group was motivated to acquire a pet. After half a year the stress task was repeated in the homes of the participants. The pet owners had their pet present during the task and showed lower blood pressure than the control group. Their cardiovascular reactivity to the stressor was lowered by half. Also, heart rate and plasma renin activity was lower in the presence of the pet.

Overall, most of these studies show that the presence of friendly animals, both familiar or unfamiliar, can effectively reduce heart rate and blood pressure or buffer increases in these parameters in anticipation of a stressor. These effects may even be stronger with one’s own pet.

### Effects on anxiety and pain

#### Reduction of fear and anxiety and promotion of calmness

Several studies investigated whether animal contact can reduce fear and anxiety elicited by a stressor. Shiloh et al. (2003) first showed participants a live tarantula spider and indicated that they might be asked to hold it later on. Participants were randomly assigned to five groups, and instructed to pet either a live rabbit, a live turtle, a toy rabbit, a toy turtle, or to just rest. Only petting a live animal, but not a toy animal reduced self-reported anxiety. Therefore, not the physical activity of petting *per se* caused the effect. Similarly, Barker et al. (2003a) investigated self-reported anxiety in psychiatric patients before electroconvulsive therapy. One group interacted with an animal for 15 min while the control group read magazines. Interacting with the animal significantly reduced anxiety and fear. In contrast, Straatman et al. (1997) did not find a significant effect on self-reported anxiety in male students during a stressful speech task, when comparing a group in the presence of an unfamiliar dog with a group without a dog present.

In a few studies the effect of animal presence or contact on self-reported anxiety in humans in the absence of a specific stressor was investigated. Cole et al. (2007) compared the effects of a 12-min visit with a dog or without a dog, with conditions of normal care in adult patients who had been hospitalized due to heart failure (which, however, could be interpreted as a natural stressor). Anxiety was reduced most in the presence of the visiting dog. In undergraduate students a comparison of reading quietly, reading aloud, and interacting with a dog (Wilson, 1991) did not show a significant effect of the animal contact on anxiety when compared to reading quietly. Also Barker and Dawson (1998) found no statistically significant difference in anxiety between AAT in comparison to therapeutic recreation in psychiatric inpatients. However, anxiety decreased from the beginning to the end of the animal-assisted session, while this was not the case in the control condition. In a study by Lang et al. (2010) patients with acute schizophrenia reported less anxiety after a clinical interview, when it was conducted in the presence of a friendly dog than without. Berget et al. (2011) compared effects of a 12-week intervention program with farm animals in psychiatric patients with various diagnoses with a control group and found a decrease in anxiety, however, not directly after the intervention, but 6 months later.

As mentioned above, aquaria have limited potential in HAI. Still, Barker et al. (2003b) showed that in a waiting room, an aquarium can reduce anxiety in psychiatric inpatients before a scheduled electroconvulsive therapy. Edwards and Beck (2002) documented a higher food intake and weight gain, and a reduced requirement of nutritional supplementation in patients with Alzheimer’s Disease after the introduction of an aquarium into the facility. This finding may be interpreted as an increase in calmness, since Alzheimer patients are often agitated and confused and this interferes with food intake, leading to problems with malnutrition and weight loss.

A similar effect of a friendly animal on calmness, the opposite of anxiety, was documented by a study by Crowley-Robinson et al. (1996). The elderly residents of a nursing home with a resident dog, reported less tension and confusion in comparison to residents of a home without a dog. Also Perkins et al. (2008) and Filan and Llewellyn-Jones (2006) concluded from their reviews that dog-assisted therapy reduces restlessness in elderly patients with dementia.

Overall, the majority of studies points to a positive effect of interactions with and observation of animals on self-reported anxiety and calmness, in particular under stress-prone conditions.

#### Effects on perception of pain

Unfortunately, none of the few existing studies on effects of animal contact on human pain management met our inclusion criteria. However, reports point at a possible positive effect, indicating a reduced use of pain medication, especially in nursing homes and homes for the elderly (e.g., Darrah, 1996) with the presence of pets.

### Effects on learning

Little research addresses animals’ positive effects on learning in children. However, there is some indirect evidence that animals could positively affect the preconditions for learning. In a series of studies, Gee and colleagues investigated the effect of the presence of a dog on children performing different tasks. A group of developmentally delayed and a group of normally developed children performed faster in a motor skill task with the same accuracy when a dog was present than when no dog was present (Gee et al., 2007). According to the authors, one explanation could be that the dog served as an effective motivator, another that the presence of the dog led to increased relaxation and a reduction of stress during execution of the task thus increasing speed of performance. Also pre-school children with and without language impairments adhered to instructions during an imitation task better in the presence of a dog than in the presence of a toy dog or a human (Gee et al., 2009). They needed fewer prompts (as an indicator of concentration) for a memory task in the presence of a dog, while they needed the most prompts in the presence of another human (Gee et al., 2010b). Furthermore, in a match-to-sample task pre-school children made fewer errors, such as irrelevant choices, in the presence of a friendly dog in comparison to the presence of a stuffed toy dog or a human (Gee et al., 2010a). In line with these results, Kotrschal and Ortbauer (2003) found that children paid more attention to the teacher when a dog was present in the classroom and overall, we propose that some of the found effects of Gee and colleagues could also be based on increased social attention toward the experimenter in the dog condition.

Currently, there is no direct evidence that animals can promote learning in humans, but the presence of a dog in an educational setting seems to support concentration, attention, motivation, and relaxation reflecting reduction of high stress levels which inhibit effective learning and performance. Also, the presence of a dog creates a pleasant social atmosphere, which is known to be an essential component for optimal executive functioning (Diamond and Lee, 2011), which represents a precondition for learning.

### Effects on human health and restoration

Already in the 1980s researchers tested the idea that pet ownership is good for the owner’s mental and physical health. However, many of these studies suffered from the problem of confounding variables, such as the individual’s health condition which may influence the decision to get a pet. Such studies, some of them large surveys with thousands of participants, were conducted by Garrity et al. (1989); Raina et al. (1999); Parslow et al. (2005); Siegel (1990); Stallones et al. (1990), and Winefield et al. (2008). Their findings generally suggest that companion animal owners have better health than non-animal owners, as indicated by medical markers such as cholesterol levels, or indirectly, via the frequency of doctor visits. However, these correlative studies do not allow making a causal connection between pet ownership and health.

#### Original studies on health effects of companion animals

Headey and his colleagues investigated health effects of pet ownership in several surveys with large and representative samples, while statistically controlling for most confounding variables and thus allowing for a more causal interpretation of pet ownership and health. In over 1000 adult Australians Headey (1999) found that dog and cat owners paid fewer annual doctor visits and were less likely to take medication for sleeping problems than non-pet owners. In a survey with over 3000 female participants between 25 and 40 years of age from Chinese cities, Headey et al. (2008) report that dog owners, who comprised half of the sample, had higher self-reported fitness and health, exercised more frequently, slept better, saw their doctors less frequently, and took fewer days off from work than comparable non-dog owners.

Another approach focuses on certain sub-populations with a well-defined social or health condition, which can contribute to a better comparability of pet-owners and non-owners with respect to many confounding variables. In their sample of empty-nesters, i.e., families in which the children have left home, Na and Richang (2003) found that pet-owning couples showed better mental and physical health than those not owning a pet. From a longitudinal survey in Australia and Germany in which the same group was questioned a second time after several years, Headey and Grabka (2007) reported that the healthiest group in both countries was the one who continuously owned a pet. They reported 15% fewer annual doctor visits than non-owners, while the influence of gender, age, marital status, income, and other variables associated with health was statistically controlled. Persons who never had a pet or ceased to have one were less healthy.

In children with cerebral palsy a 10-week therapeutic riding program had no effect on general health and quality of life in comparison to a control group (Davis et al., 2009). However, indirect indicators of mental health, self-efficacy and coping ability, had significantly improved 6 months after the end of a 12-week intervention with farm animals in psychiatric patients in comparison with a control group, while directly after the intervention no significant differences between the two groups were found (Berget et al., 2008). In elderly patients with schizophrenia, AAT in weekly 4 h sessions over the course of 12 months improved adaptive functioning, which is frequently used as a global indicator of mental health and describes a person’s ability to take care of him- or herself and to effectively interact with society (Barak et al., 2001).

#### Reviews on health effects of human-animal interactions

In two review articles on pet ownership and human health, Wells (2007, 2009) concluded that there is evidence supporting the prophylactic and therapeutic value of companion animals to humans without providing direct evidence for a causal association. Nimer and Lundahl (2007) concluded from their meta-analysis of 49 studies that animal-assisted interventions (excluding pet ownership or mere animal-assisted activities) showed positive health effects, often with medium effect sizes for persons of all ages with regard to emotional well-being, medical conditions, behavioral problems, and symptoms of autistic-spectrum disorder.

#### Cardiovascular disease and human-animal interaction

Evidence for the effects of HAI on cardiovascular diseases was gained from the following studies: Headey (1999) found that dog and cat owners were less likely to take medication for heart problems than non-pet owners. Friedmann and Thomas (1998) investigated 1-year-survival in a group of several hundred patients with acute myocardial infarction. High social support and owning a dog, but not a cat, predicted survival after 1 year. In their review of research on “pet therapy,” Giaquinto and Valentini (2009) come to the conclusion that there is consistent evidence for a protective effect of pet ownership against cardiovascular risk.

#### Effects of human-animal interaction on the immune system

Only one study assessing parameters of the immune system met our inclusion criteria. Charnetski et al. (2004) reported a significant increase in salivary immunoglobulin A (IgA), an indicator of good immune system functioning, in college students after stroking a live dog in comparison to stroking a stuffed dog or sitting quietly for 18 min.

### Summary of effects of human-animal interaction

The studies reviewed here clearly indicate the following positive effects of HAI in several different domains and in humans of different age groups, with and without special medical, or mental health conditions:

– improvement of social attention, behavior, interpersonal interaction, and mood
– reduction of stress-related parameters such as cortisol, heart rate, and blood pressure
– reduction of self-reported fear and anxiety
– improvement of mental and physical health, especially cardiovascular health.
  Limited evidence or very few publications exist for positive effects of HAI on:
– reduction of stress-related parameters such as epinephrine and norepinephrine
– improvement of immune system functioning
– improved pain management
– increased trustworthiness of and trust toward other persons
– reduced aggression
– enhanced empathy and
– improved learning.

We propose that most of these effects of HAI may be mediated via the OT system and that the activation of this system represents the mechanism underlying these effects. Exceptions are the studies involving aquaria or aviaries which do not allow for direct contact. The calming effects of observing fish or birds are likely mainly based on other mechanisms than the activation of the OT system.

It needs to be mentioned here that obviously the reported studies worked with an optimal setting, i.e., persons who voluntarily participate and can be assumed to have an at least neutral to positive attitude toward the involved species and no fear of the involved animals. Stress-reducing and calming effects of e.g., dogs cannot be expected in persons with a dog phobia. This self-selection limits the generalization to the entire population, however, it seems plausible to assume that the reported effects can be found in persons with a similar neutral to positive attitude toward animals. Also familiarity with the animal species might have an influence in this respect. Furthermore, also the involved animals, particularly if they do not belong to the participants, usually meet high standards, meaning they are obedient, calm, friendly, healthy, and well-socialized with humans with special needs and diseases. Therefore not all results from experimental settings can be generalized to every privately kept companion animal, or at least the positive effects might be blunted by a pet’s behavior or health problems.

## The Oxytocin System

The peptide hormone oxytocin (OT) is produced in the hypothalamus and released into the circulatory system and the brain in response to sensory stimulation via a network of OT-containing nerves (Landgraf and Neumann, 2004; Ross et al., 2009), e.g., during breastfeeding, labor, sex, but also touch, warmth, and stroking, usually in the context of trusting relationships (for reviews see Uvnäs-Moberg, 2003; Insel, 2010). Many physiological, psychological and behavioral functions are modulated via OT, as has been shown via experimental administration of OT in animals and humans.

### Effects on social interaction

Among the acute effects of OT is the stimulation of social interaction. It increases eye contact, empathy, face memory, trust, social skills, positive self-perception, and generosity and decreases depression (Heinrichs et al., 2003; Kosfeld et al., 2005; Ohlssson et al., 2005;Zak et al., 2005, 2007; Domes et al., 2007; Guastella et al., 2008; Jonas et al., 2008; Savaskan et al., 2008; Rimmele et al., 2009; Cardoso et al., 2011). Also, it counteracts aggression (Petersson et al., 1998) and improves learning by conditioning (Björkstrand et al., 1996;Petersson et al., 1996, 1999; Uvnäs-Moberg et al., 2000). OT promotes maternal care behavior (Pedersen et al., 1982; Fahrbach et al., 1984) and bonding to the offspring (Kendrick et al., 1986, 1997; Keverne and Kendrick, 1992) as well as pair bonding (Carter et al., 1995).

### Anti-stress effects

Furthermore, OT has anti-stress effects. It decreases glucocorticoid (i.e., stress hormone) levels in humans and non-human animals (Legros et al., 1988; Petersson et al., 1999; Neumann et al., 2000), in particular in response to social stressors (Heinrichs et al., 2003; Kirsch et al., 2005). When administered intracerebroventricularly, OT decreases blood pressure for several hours (Petersson et al., 1999) as well as heart rate (Dreifuss et al., 1988), and increases peripheral cutaneous circulation and skin temperature (Petersson et al., 1999).

### Effects on anxiety, pain, and immune system

Oxytocin increases pain thresholds and has an anti-inflammatory effect in rats (Petersson et al., 1996, 2005). It has also an anxiolytic effect (Uvnäs-Moberg et al., 1994; Neumann et al., 2000; Amico et al., 2004; Jonas et al., 2008; Guastella et al., 2009), in particular in relation to social threats (Kirsch et al., 2005).

### Effects on health and restoration

Furthermore, OT is associated with increases in the function of the parasympathetic nervous system controlling the endocrine system of the gastrointestinal tract, which is linked to an enhanced digestive function and growth and restoration (Widstrom et al., 1988; Uvnäs-Moberg, 1989, 1994).

As this short overview of OT-mediated effects shows there is a major overlap of effects of HAI and OT and the following research shows that indeed HAI may activate the OT system in humans.

## Effects of HAI on Oxytocin

Odendaal (2000); Odendaal and Meintjes (2003) documented a significant increase of plasma OT, as well as prolactin, phenylacetic acid, and dopamine, in both, humans and dogs after 5 to 24 min of stroking a dog. Interaction with one’s own dog resulted in a stronger effect than stroking an unfamiliar dog. This indicated that the increase in OT depends on the quality of the human-animal relationship – the closer the relationship, the more OT is released via the positive interaction including physical contact.

Miller et al. (2009) studied changes of plasma OT via interaction with the person’s own pet-dog, in men and women after coming home from work who had been separated from their dog during the day. Two conditions, interacting with the dog and reading quietly in the absence of the dog were compared. Interacting with the dog led to an increase in plasma OT in women, but not men, while this was not observed in the reading condition. In both conditions, men even showed a drop in OT levels. However, OT levels decreased more in the control condition, pointing toward a counteracting effect of interacting with the dogs on the OT system also in men.

In their study of female dog owners, who were instructed to stroke and talk to their own male dogs for 3 min in a laboratory setting, Handlin et al. (2011) found a significant increase in plasma OT in both women and dogs, but not in a control group without animal interaction.

Nagasawa et al. (2009) employed urinary OT to assess the effect of 30 min of interaction between dogs and their owners, particularly the duration of friendly gazes from the dogs to the owners. In a control condition lasting for 30 min, owners were instructed not to look at their dogs directly. Participants were divided into owners who were less attached and received a shorter duration of gaze from their dogs and owners who received a longer duration of gaze from their dog and reported a higher degree of attachment to them. In the normal interaction condition, longer gaze was linked to higher OT levels in the owner, while this was not the case in the control condition without eye contact.

It needs to be mentioned that measuring urinary OT is not generally accepted as a valid approach to assess peripheral OT. Indeed, Feldman et al. (2011) found no significant correlation between urinary OT and plasma or salivary OT, but a moderate correlation of the latter two. The techniques for assessing OT levels differ between the studies reported above and thus did not necessarily measure exactly the same, which needs to be kept in mind when comparing their results (Uvnäs-Moberg et al., 2011).

Overall, the studies suggest that social interaction of humans and dogs may lead to an increase in OT levels in both, the human and the dog. Physical contact and the relationship between owner and dog seem to play an important role in this respect.

## Conclusion

Research on direct OT effects in HAI is still rare. But the existing evidence clearly points at the potential of interactions with animals, especially one’s own pet-dog, to increase OT levels in humans. We propose that the release of OT via contact with animals may contribute to explain many of the effects of HAI documented by the studies included in our review. There is also much indirect evidence on a potential link of HAI effects and the OT system. In fact, the effects of OT and of HAI largely correspond. Both, HAI and OT, were found to promote social interaction, to reduce stress and anxiety, and to enhance human health. OT is released via eye contact, but in particular, via pleasant tactile interactions which seem to play a major role for the OT-mediated decrease of stress levels. Oxytocin effects may be triggered in response to single meetings with animals, but stable relationships with animals such as pet ownership will be linked to more potent and long lasting effects due to repeated exposure to OT. In addition, the bond between human and animal may contribute to OT release and oxytocin mediated effects. HAI effects are stronger with a familiar dog in comparison to an unfamiliar dog. That the OT system plays a major role in social bonds has been documented by several studies (e.g., Carter, 1998; Carter and Keverne, 2002; Bales et al., 2007).

Overall, we propose that the reduction of subjective psychological stress (fear, anxiety) due to animal contact, as well as the dampening of physiological stress parameters in connection with activation of the OT system represent a core mechanism in explaining many of the positive effects of HAI. These mechanisms have also be linked to other constructs such as e.g., attachment theory, biophilia (Wilson, 1984), social support theories (Wills, 1991), the polyvagal theory (Porges, 1995, 2007, 2009) and the social baseline model (Coan, 2008, 2010, 2011) by a large body of studies which focus on stress regulation via social support (Wills, 1991) and via bonds within one’s own species Julius et al. (2012). The evidence reviewed here clearly indicates that these mechanisms may also apply to some extent to HAI. Also, while we believe that the activation of the OT system is a core mechanism in explaining the majority of reported effects of HAI, we are aware that it cannot explain all effects and that other mechanisms, physiologically as well as psychologically based, are involved. Frequently cited factors in research on HAI are e.g., the unconditional love and acceptance which animals provide to clients of animal-assisted interventions and their owners and the social catalyst effect which might be initially based rather on biophilia than the activation of the OT system. Biophilia can be described as an interest in nature and animals and in seeking a connection with them (Wilson, 1984). However, the activation of the OT system via HAI as theoretic approach allows the integration of many different research findings due to the large body of research on OT in animal or human research which can serve as foundation, which no other theory has been able to achieve until now.

## Conflict of Interest Statement

The authors declare that the research was conducted in the absence of any commercial or financial relationships that could be construed as a potential conflict of interest.
